# Use of liposomal anfotericin B in diseminated cutaneous leishmaniasis caused by *Leishmania braziliensis* in a pediatric patient with Down syndrome

**DOI:** 10.17843/rpmesp.2023.401.11481

**Published:** 2023-03-22

**Authors:** Aidé Sandoval-Juárez, Nyshon Rojas-Palomino, Lenka Kolevic Roca, Graciela Pilares-Barco, Jorge Cuadros-Castro, Roger Araujo-Castillo

**Affiliations:** 1 National Referral Laboratory for Leishmaniasis, Instituto Nacional de Salud, Lima, Peru. National Referral Laboratory for Leishmaniasis Instituto Nacional de Salud Lima Peru; 2 Instituto Nacional de Salud del Niño, Lima, Peru. Instituto Nacional de Salud del Niño Lima Peru; 3 National Center for Public Health, Instituto Nacional de Salud, Lima, Peru. National Center for Public Health Instituto Nacional de Salud Lima Peru

**Keywords:** Down Syndrome, Cutaneous Leishmaniasis, Amphotericin B, Leishmania braziliensis

## Abstract

We present a case of disseminated cutaneous leishmaniasis with extensive manifestation in a pediatric patient with Down syndrome. The case was confirmed by parasitological and immunological tests. The species was identified as *Leishmania (Viannia) braziliensis* by polymerase chain reaction-restriction fragment length polymorphisms (PCR-RFLP). The immune deficit that occurs as part of Down syndrome may have been the reason for the aggressive and prolonged clinical manifestations as well as the poor response to stibogluconate and deoxycholate amphotericin. The patient was treated with liposomal amphotericin B and at the end of therapy, showed clinical improvement of the lesions. This report highlights the challenges of the diagnosis and treatment of cutaneous leishmaniasis in immunosuppressed pediatric patients, especially under difficult social, economic and geographic conditions. Leishmaniasis should be considered as a differential diagnosis when treating atypical chronic dermatologic ulcers; the use of liposomal amphotericin in immunocompromised patients should also be considered in these cases.

## INTRODUCTION

Leishmaniasis is a disease distributed throughout the world ^1^, caused by more than 20 species of *Leishmania*^2^. According to the World Health Organization, this disease can be classified by its clinical presentation as cutaneous, mucosal and visceral leishmaniasis. Cutaneous leishmaniasis is subclassified as localized cutaneous, diffuse cutaneous (DCL) and disseminated leishmaniasis (DL); mucosal and visceral leishmaniasis are lethal, if not diagnosed and treated in a timely manner ^3^. The cutaneous form presents ulcerative, nodular or infiltrative lesions and, depending on its location, can cause psychological repercussions, affecting self-esteem and social development ^4^.

The different cutaneous varieties are caused by the interaction of factors such as the *Leishmania* species and the patient’s immune response ^1^. Cases may worsen, present atypical forms and have inadequate response to treatment, particularly in patients with alterations of the immune system like the human immunodeficiency virus (HIV) ^5^^,^^6^, patients receiving medication such as corticosteroids ^7^ or due to genetic conditions such as Down syndrome ^8^. In the present report, we describe the case of a five-year-old patient with Down syndrome and disseminated cutaneous leishmaniasis caused by *Leishmania (Viannia) braziliensis*.

## CASE REPORT

Five-year-old female patient with a history of Down syndrome, from the province of Quillabamba, department of Cusco, an Amazon jungle area endemic for leishmaniasis. Her father reported that, at the age of one year, the child began to develop papular lesions on the face, which slowly increased in size. One year after their appearance, the lesions ulcerated near the malar and mandibular region on the right side of the face. The patient was transferred and diagnosed with cutaneous leishmaniasis at the Regional Hospital of Cusco in June 2016, where she was treated with amphotericin B deoxycholate at a dosage of 7 mg every 24 h for 37 days (cumulative dose of 259 mg); she showed little improvement after treatment.

In 2017, the patient was admitted to a different level III hospital in Cusco due to multiple lesions on the face, buttock, lower limbs, elbow, right hand, respiratory distress and laryngeal stridor. Amphotericin B deoxycholate was administered at a dose of 9 mg every 24 hr for five months (cumulative dose of 952.4 mg). She also received sodium stibogluconate for 25 days, imiquimod daily and miltefosine at 30 mg/day for two months (weight: 12 kg). Response to treatment was poor, with persistent ulcers on the right elbow and face, laryngeal stridor and dysphonia; despite treatment, new lesions appeared on the left thigh. Subsequently, the patient was lost to follow-up, although in 2018, she received 10 mg/day of prednisone for three months at a healthcare facility.

**Figure 1:**
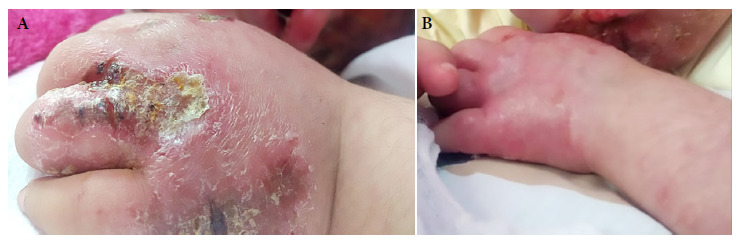
Left upper limb of a five-year-old patient with Down syndrome and disseminated cutaneous leishmaniasis (A) Before treatment with liposomal amphotericin B (B) After receiving 11 doses of liposomal amphotericin B (3 mg/kg/dose).

**Figure 2:**
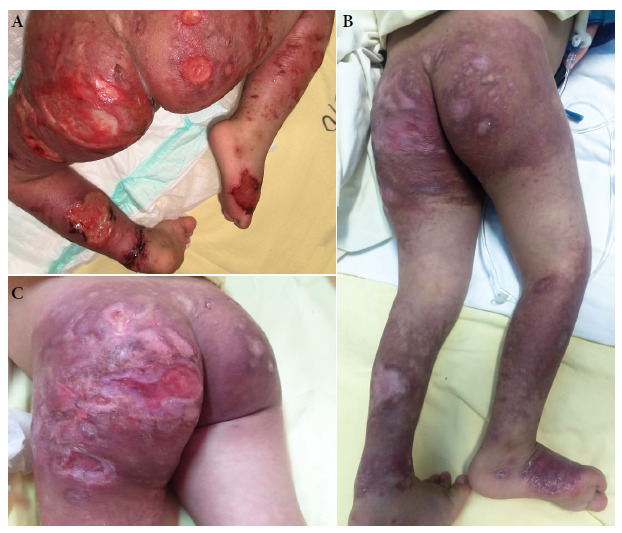
Lower limbs and gluteal area of a five-year-old patient with Down’s syndrome and disseminated cutaneous leishmaniasis (A) Before treatment with liposomal amphotericin B (B) After receiving 11 doses of liposomal amphotericin B (3 mg/kg/dose) (C) After receiving 15 doses of liposomal amphotericin B (3 mg/kg/dose).

On January 2019, the patient was admitted again to the Regional Hospital of Cusco and then referred to the National Institute of Child Health in Lima because of the increasing size of the lesions (Figures 1A and 2A). Multiple ulcers with infiltrated raised borders were found during physical examination, these lesions were distributed on the buttocks, elbows, arms, legs and chin, with diameters ranging from 1 cm in the arms and legs to 8 cm in the buttocks. Erosions in the oral mucosa of 0.5 cm, subcostal retractions and diffuse rhonchi in both lungs were also found. No other signs and symptoms were found during examination.

Skin biopsy revealed hyperkeratosis, irregular epidermal hyperplasia, fibrous dermis with predominantly dense, lymphohistiocytic, inflammatory infiltrate with some plasma cells, and histiocytes with abundant structures suggestive of *Leishmania* spp. amastigotes. Amastigote forms were found during direct microscopic examination (smear test); this procedure was carried out at the National Referral Laboratory of Leishmaniasis of the National Institute of Health since the periodic acid-Schiff stain (PAS) and the differentiation group 1A (CD1A) were negative. The Montenegro intradermal reaction test (IDRM) was positive, with a 7 × 8 mm induration at 48 hrs. Anti-Leishmania IgG antibodies were detected in the patients’ serum by indirect immunofluorescence, with a positive titer of 1/160.

**Figure 3:**
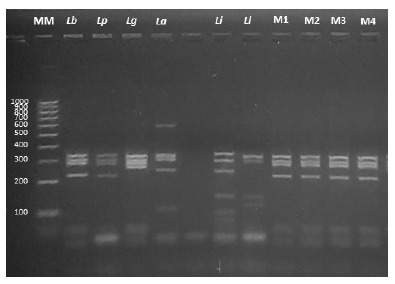
PCR-RFLP electrophoresis profile of the *Hsp70* using HaeIII enzyme. MM: Molecular marker. Reference strains: *Lb: Leishmania (V.) braziliensis, Lp: Leishmania (V.) peruviana, Lg: Leishmania (V.) guyanensis, La: Leishmania (L.) amazonensis, Li: Leishmania (L.) infantum, Ll: Leishmania (V.) lainsoni*. M1 and M2: DNA samples from slides with tissue smears. M3 and M4: DNA samples from lancet.

**Figure 4:**
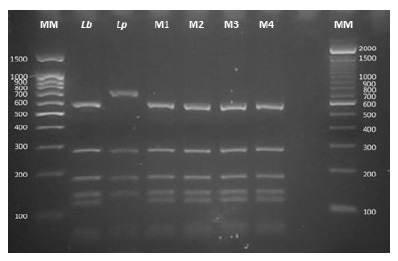
PCR-RFLP electrophoresis profile of the *Hsp70* using RsaI enzyme. MM: Molecular marker. Reference strains: *Lb: Leishmania (V.) braziliensis, Lp: Leishmania (V.) peruviana*. M1 and M2: DNA samples from slides. M3 and M4: DNA samples from lancet.

Genomic DNA samples extracted from lesion tissue revealed that the disease was caused by *Leishmania* (*Viannia*) *braziliensis.* Using a polymerase chain reaction-restriction fragment length polymorphisms (PCR-RFLP) technique, the region of the gene encoding the heat shock protein (Hsp70) was amplified and then subjected to an enzymatic digestion process with HaeIII and RsaI, according to the methodology described by Montalvo *et al*. in 2012 ^9^ (Figures 3 and 4). Flow cytometry analysis was carried out at the National Referral Center for Allergy, Asthma and Immunology (CERNAAI) and at the Immunology Laboratory of the Institute of Tropical Medicine of the Peruvian Cayetano Heredia University. Laboratory tests showed the following: total leukocytes: 4600 cells/µL, CD3/CD4 lymphocytes: 258 cells/µL (normal range: 500-2700), regulatory T lymphocytes: 14 cells/µL (normal range: 35-140), T Helper CD4 lymphocytes: 251 cells/µL (normal range: 1500-5000 cells/µL), CD8 Cytotoxic T cells: 627 cells/µL (normal range: 500-1600 cells/µL), B lymphocytes: 50 cells/µL (normal range: 600-3000 cells/µL), and Natural Killer (NK) lymphocytes: 195 cells/µL (normal range: 100-1300 cells/µL).

The patient was diagnosed with DL and treatment started with liposomal amphotericin B, 3 mg/kg/day intravenously every 5 to 6 days, she received 15 doses. Dermal infections were also treated. Patients’ condition improved after the 11th dose (Figures 1B and 2B), up until the 15th dose (Figure 2C).

## DISCUSSION

We report the case a pediatric patient with Down syndrome who presented an aggressive clinical form of leishmaniasis, consistent with DL, which started during the first year of the child´s life, following a chronic and persistent course without improvement for four years, despite having received multiple anti-leishmanial treatments with amphotericin B deoxycholate, imiquimod and miltefosine. In addition to the underlying disease and chronic course of DL, the rural area with limited health resources in which the patient lived interfered with treatment.

In Peru, the incidence of DL is unknown; however, worldwide, this form of leishmaniasis represents less than 2% of all cutaneous leishmaniasis cases ^10^^,^^11^. Despite having similar characteristics to DCL, the clinical presentation of DL helps differentiating between the two forms. DL is mainly caused by *Leishmania (Viannia) braziliensis*; however, there are also reports of other species of DL by the subgenus *Viannia*. In contrast, DCL is mostly caused by the subgenus *Leishmania*^12^^,^^13^, although there are rare reports caused by other species ^14^.

DL usually progresses quickly, and up to 25% of cases occur post-treatment, the lesions are pleomorphic, acneiform, nodular, papular or ulcerative, and spread throughout the body, even affecting the mucous membranes ^15^^-^^17^. On the other hand, DCL is a chronic disease that presents nodules and papules that infiltrate the skin, but it doesn’t produce ulcers and does not affect mucous membranes ^15^^,^^17^^,^^18^. Likewise, DL’s response to the IDRM test is positive in 67 to 83% of the cases ^12^^,^^15^^,^^16^^,^^18^, while it is always negative for DCL ^12^^,^^16^^,^^19^.

Treatment for both clinical forms is usually longer than usual, with lower response rates, and sometimes requires several complete courses of treatment due to relapses ^12^^,^^16^^,^^18^^,^^20^, especially in individuals with particular immunological conditions, as in the present case. The patient did not only have Down syndrome, which decreases the cellular responsiveness of the immune system, but also received corticosteroids for a long time prior to her last hospitalization. It is important to note that this patient had active lesions for four years without resolution of symptoms despite having received full courses of anti-leishmanial therapy including pentavalent antimonials, oral miltefosine and amphotericin B deoxycholate. On the other hand, high doses of liposomal amphotericin B were used during an extended period of time, which showed improvement of the lesions.

Liposomal amphotericin B is an alternative for the treatment of cutaneous leishmaniasis and has proven to be as effective as deoxycholate amphotericin in multiple studies, but with fewer adverse events and a better safety profile. North American guidelines for the management of leishmaniasis state that it is better tolerated than deoxycholate, and less nephrotoxic. Moreover, there are reports of the successful use of liposomal amphotericin B in complex forms of cutaneous leishmaniasis; it has also been described in cases of DL in pediatric patients, similar to the case presented in this report.

This report illustrates the challenges of both diagnosis and treatment of cutaneous leishmaniasis in immunosuppressed pediatric patients, especially in an environment with social, economic, and geographic barriers to access to health services. Such barriers may have contributed to delays in diagnosis, as well as treatment interruptions, and lack of adequate follow-up.

In conclusion, leishmaniasis should be considered during differential diagnosis when treating atypical chronic skin ulcers, even those with a disseminated pattern in different anatomical areas, especially in immunosuppressed patients with epidemiological suspicion. Likewise, it is important to highlight the importance of identifying the *Leishmania* species, especially in aggressive or atypical forms of the disease, this information can help predicting the prognosis of the disease, and establishing personalized clinical management. Finally, it is important to highlight the therapeutic effectiveness of liposomal amphotericin B in immunocompromised patients who require a more aggressive and prolonged treatment than usual.
