# Supplementary material for: Use of liposomal anfotericin B in diseminated cutaneous leishmaniasis caused by Leishmania braziliensis in a pediatric patient with Down syndrome
Source: Rev Peru Med Exp Salud Publica. 2023 Mar 22;40(1):94–8. doi: 10.17843/rpmesp.2023.401.11481 (PMC10953639; doi:10.17843/rpmesp.2023.401.11481)
Supplement: Translation

# Translation: Uso de anfotericina B liposomal en leishmaniasis cutánea diseminada causada por *Leishmania braziliensis* en un paciente pediátrico con síndrome de Down

**Authors:** Aidé Sandoval-Juárez, Nyshon Rojas-Palomino, Lenka Kolevic Roca, Graciela Pilares-Barco, Jorge Cuadros-Castro, Roger Araujo-Castillo

**Affiliations:** 1 Laboratorio de Referencia Nacional de Leishmaniasis, Instituto Nacional de Salud, Lima, Perú.; 2 Instituto Nacional de Salud del Niño, Lima, Perú.; 3 Centro Nacional de Salud Pública, Instituto Nacional de Salud, Lima, Perú.

**Keywords:** Síndrome de Down, Leishmaniasis, Anfotericina B, Leishmania braziliensis

## Abstract

Se presenta un caso de leishmaniasis selvática cutánea diseminada con manifestación extensa en una paciente pediátrica con síndrome de Down. El caso se confirmó a través de estudios parasitológicos e inmunológicos, mientras que la identificación se realizó mediante la técnica de reacción en cadena de la polimerasa-polimorfismos de longitud de fragmentos de restricción (PCR-RFLP, por sus siglas en inglés), determinándose la especie como *Leishmania (Viannia) braziliensis*. La manifestación clínica agresiva y prolongada con poca respuesta a estibogluconato y anfotericina desoxicolato pueden deberse al déficit inmunológico que se presenta como parte del síndrome de Down. La paciente eventualmente recibió tratamiento con anfotericina B liposomal y al término de la terapia, mostró mejoría clínica de las lesiones. El presente reporte ilustra los desafíos tanto de diagnóstico como tratamiento de leishmaniasis cutánea en pacientes pediátricos inmunosuprimidos, especialmente en un entorno de difícil acceso social, económico y geográfico, a los servicios de salud. Se recomienda considerar a la leishmaniasis en el diagnóstico diferencial cuando se atienda ulceras crónicas dermatológicas atípicas; así como tener en cuenta el uso de anfotericina liposomal en pacientes inmunocomprometidos.

## INTRODUCCIÓN

La leishmaniasis es una enfermedad ampliamente distribuida en el mundo ^1^, causada por más de 20 especies de *Leishmania*^2^*.* Según la Organización Mundial de la Salud, esta enfermedad puede clasificarse según la presentación clínica como leishmaniasis cutánea, a su vez subclasificada como cutánea localizada, cutánea difusa (LCD) y diseminada (LD); la leishmaniasis mucosa y la forma visceral son letales, si no son diagnosticadas y tratadas oportunamente ^3^. La forma cutánea se presenta como lesiones ulcerativas, nodulares o infiltrativas y dependiendo de su localización puede generar repercusiones a nivel psicológico, afectando su autoestima y desenvolvimiento social ^4^.

Las diferentes variedades cutáneas ocurren debido a la interacción de múltiples factores, principalmente entre la especie de *Leishmania* involucrada y la respuesta inmune del paciente ^1^. En este contexto, existe la posibilidad de agravarse, presentar formas atípicas e inclusive tener una inadecuada respuesta al tratamiento, especialmente en aquellos pacientes que presenten alteraciones del sistema inmune, como son las personas viviendo con el virus de inmunodeficiencia humana (VIH) ^5^^,^^6^, bajo la administración de ciertos fármacos como corticoides ^7^ o por condiciones genéticas como el síndrome de Down ^8^. En el presente reporte, se describe el caso de una paciente de cinco años con síndrome de Down que presentó la forma cutánea diseminada causada por *Leishmania (Viannia) braziliensis.*

## REPORTE DE CASO

Paciente mujer de cinco años, natural y procedente de la provincia de Quillabamba, departamento del Cusco, una zona de selva amazónica endémica para leishmaniasis, con antecedente de síndrome de Down. El padre refiere que, a la edad de un año, la menor inicia con la aparición de lesiones papulares en rostro, los cuales presentaron un incremento lento en sus dimensiones. Un año después de su aparición, las lesiones se ulceraron a la altura de la región malar y mandibular en hemicara derecha. La menor fue traslada y diagnosticada como leishmaniasis cutánea en el Hospital Regional del Cusco en junio de 2016, donde recibió como tratamiento anfotericina B desoxicolato a la dosificación de 7 mg cada 24 h por 37 días (dosis acumulada de 259 mg), al término del cual, se evidenció poca respuesta.

En 2017, es ingresada a otro hospital nivel III de Cusco por presentar múltiples lesiones en cara, glúteo, miembros inferiores, codo, mano derecha, dificultad respiratoria y estridor laríngeo. Se administró anfotericina B desoxicolato a una dosis de 9 mg cada 24 hrs por cinco meses (dosis acumulada de 952,4 mg), estibogluconato de sodio por 25 días, imiquimod interdiario y miltefosina a 30 mg/día por dos meses (peso: 12 kg). La respuesta clínica fue pobre, persistiendo las úlceras de codo derecho y rostro, estridor laríngeo y disfonía; a pesar del tratamiento, aparecieron nuevas lesiones en muslo izquierdo. Posteriormente, se perdió el seguimiento del paciente, aunque en 2018, recibió prednisona a la dosis de 10 mg/día durante tres meses en un establecimiento de salud.

En enero del 2019, la paciente es ingresada nuevamente al Hospital Regional de Cusco y luego referida al Instituto Nacional de Salud del Niño en Lima, la capital del país por aumento del tamaño de las lesiones (Figuras 1A y 2A). Al examen físico, se encuentran múltiples úlceras con bordes elevados infiltrados, distribuidas en glúteos, codos, brazos, piernas, mentón, con diámetros de 1 cm en extremidades a 8 cm en glúteos; erosiones en la mucosa oral de 0,5 cm; tiraje subcostal y roncantes difusos en ambos pulmones. El resto del examen no fue contributorio.

**Figura 1:** Miembro superior izquierdo de paciente de cinco años con síndrome de Down y leishmaniasis cutánea diseminada. (A) Previo al tratamiento con anfotericina B liposomal. (B) Luego de recibir 11 dosis de anfotericina B liposomal (3 mg/kg/dosis).

**Figura 2:** Miembros inferiores y zona glútea de paciente de cinco años con síndrome de Down y leishmaniasis cutánea diseminada. (A) Previo al tratamiento con anfotericina B liposomal. (B) Luego de recibir 11 dosis de anfotericina B liposomal (3 mg/kg/dosis). (C) Luego de recibir 15 dosis de anfotericina B liposomal (3 mg/kg/dosis).

Se realizó una biopsia de piel para el estudio anatomopatológico encontrando hiperqueratosis, hiperplasia epidérmica irregular, dermis de aspecto fibrosado con denso infiltrado inflamatorio a predominio linfohistiocitario con algunas células plasmáticas, e histiocitos con abundantes estructuras sugerentes de amastigotes de *Leishmania* spp*.* Al ser la coloración de ácido periódico de Schiff (PAS) negativa y el grupo de diferenciación 1A (CD1A) negativo, se realizó el examen microscópico directo o frotis a cargo del Laboratorio de Referencia Nacional de Leishmaniasis del Instituto Nacional de Salud, resultando positivo por visualización de formas amastigotes. Asimismo, presentó un resultado positivo con un diámetro de 7 × 8 mm en la prueba de intradermorreacción de Montenegro (IDRM) a las 48 h, y detección de anticuerpos IgG anti-*Leishmania* circulantes en suero mediante la inmunofluorescencia indirecta con un título positivo de 1/160. Por el contrario, no se logró el aislamiento del parásito debido a la infección sobreagregada de las lesiones.

Se identificó como causante de la enfermedad a *Leishmania (Viannia) braziliensis,* a partir de las muestras de ADN genómico extraído del tejido de lesión. Usando una técnica de reacción en cadena de la polimerasa-polimorfismos de longitud de fragmentos de restricción (PCR-RFLP, por sus siglas en inglés), se amplificó la región del gen que codifica la proteína de choque térmico *(Hsp70)* luego sometido a un proceso de digestión enzimática con HaeIII y RsaI, según la metodología descrita por Montalvo *et al.* en 2012 ^9^ (Figuras 3 y 4). También se realizó un análisis mediante citometría de flujo en el Centro de Referencia Nacional de Alergia, Asma e Inmunología (CERNAAI) y en el Laboratorio de Inmunología del Instituto de Medicina Tropical de la Universidad Peruana Cayetano Heredia. Se reportó leucocitos totales de 4600 células/µL, linfocitos CD3/CD4 de 258 células/µL (valor normal: 500-2700), linfocitos T reguladores en cantidad de 14 células/µL (valor normal: 35-140), linfocitos T Helper CD4 con 251 células/µL (valor normal: 1500-5000 células/µL), T Citotóxicos CD8 con 627 células/µL (valor normal: 500-1600 células/µL), linfocitos B a 50 células/µL (valor normal: 600-3000 células/µL), y linfocitos Natural Killer (NK) con una población de 195 células/µL (valor normal: 100-1300 células/µL).

**Figura 3:** Perfil de electroforesis de la PCR-RFLP *Hsp70* empleando la enzima HaeIII*.* MM: Marcador molecular. Cepas de referencia: *Lb: Leishmania (V.) braziliensis, Lp: Leishmania (V.) peruviana, Lg: Leishmania (V.) guyanensis, La: Leishmania (L.) amazonensis, Li: Leishmania (L.) infantum, Ll: Leishmania (V.) lainsoni.* M1 y M2: Muestras de ADN procedentes de láminas con extendido de tejido. M3 y M4: Muestras de ADN procedentes de lanceta.

**Figura 4:** Perfil de electroforesis de PCR-RFLP *Hsp70* empleando la enzima RsaI*.* MM: Marcador molecular. Cepas de referencia: *Lb: Leishmania (V.) braziliensis, Lp: Leishmania (V.) peruviana.* M1 y M2: Muestras de ADN procedentes de láminas. M3 y M4: Muestras de ADN procedentes de lanceta.

La paciente fue catalogada como un caso de LD e inicio tratamiento con anfotericina B liposomal, 3 mg/kg/día vía endovenosa cada 5 a 6 días, por un total de 15 dosis, además de manejar las infecciones dérmicas sobreagregadas. Luego de la dosis número 11, ya se observaba una mejoría clínica (Figuras 1B y 2B); que fue notable al término de las 15 dosis recibidas (Figura 2C).

## DISCUSIÓN

El reporte corresponde a una paciente pediátrica con síndrome de Down que presentó una forma clínica agresiva, concordante con una LD, el cual inició en el primer año de la menor, siguiendo un curso crónico y persistente sin mejoría durante cuatro años, a pesar de haber recibido múltiples tratamientos anti-*leishmania* con anfotericina B desoxicolato, imiquimod y miltefosina. Además de la enfermedad de base y la evolución crónica de la LD, el tratamiento se vio afectado debido a que el paciente residía en una zona rural con limitados recursos de salud.

En Perú, se desconoce la incidencia de la LD, sin embargo, se estima que en el mundo afecta a menos del 2% de total de casos de leishmaniasis cutánea ^10^^,^^11^. A pesar de presentar características similares a la LCD, la presentación clínica de LD permite la diferenciación entre ambas formas. En el caso de la LD, es causada principalmente por *Leishmania (Viannia) braziliensis*, sin embargo, también existen reportes de otras especies del subgénero Viannia. Por el contrario, la LCD es causada principalmente por el subgénero Leishmania ^(^^12^^,^^13^, aunque hay registros inusuales causados por otras especies ^14^.

La progresión de la LD suele ser rápida, y hasta el 25% de los casos ocurren postratamiento, las lesiones son pleomórficas, de tipo acneiformes, nodulares, papulares o ulcerativas, que se diseminan a lo largo del cuerpo afectando incluso a las mucosas ^15^^-^^17^. En cambio, la LCD es crónica, no hay presencia de úlceras, apareciendo nódulos y pápulas que se infiltran en las zonas dérmicas del cuerpo, sin afectar las mucosas ^15^^,^^17^^,^^18^. Así mismo, la respuesta a la prueba de la IDRM, en la LD es positiva entre el 67 y 83% de los casos ^12^^,^^15^^,^^16^^,^^18^, mientras que para la LCD es siempre negativa ^12^^,^^16^^,^^19^.

En ambas formas clínicas, el tratamiento suele ser más prolongado de lo usual, con tasas de respuesta más bajas, requiriendo a veces varios cursos completos de tratamiento debido a la ocurrencia de recidivas ^12^^,^^16^^,^^18^^,^^20^, especialmente en personas que cuentan con condiciones inmunológicas particulares como en el presente caso. La paciente no solo tiene síndrome de Down, el cual disminuye la capacidad de respuesta celular del sistema inmune, sino que además recibió corticoides en forma prolongada antes de su última hospitalización. Es notable que, esta paciente haya tenido lesiones activas durante cuatro años sin resolución de síntomas a pesar de haber recibido cursos completos de tratamiento anti-*leishmania* incluyendo antimoniales pentavalentes, miltefosina oral y anfotericina B desoxicolato. Por otro lado, el uso de anfotericina B liposomal permitió administrar una mayor dosis de este medicamento a la paciente por un tiempo prolongado, logrando alcanzar una mejoría marcada de las lesiones.

La anfotericina B liposomal es una alternativa para el tratamiento de la leishmaniasis cutánea, que ha probado ser tan efectiva como la anfotericina desoxicolato en múltiples estudios, pero presentando menos eventos adversos y mejor perfil de seguridad. De esta manera, la guía norteamericana de manejo de la leishmaniasis menciona que es mejor tolerada que el desoxicolato, y menos nefrotóxica. Mas aún, hay reportes exitosos de su uso en formas complejas de leishmaniasis cutánea e incluso está descrito su uso para el manejo de la LD en pacientes pediátricos, similar al caso presentado en este reporte.

El presente reporte ilustra los desafíos tanto de diagnóstico como de tratamiento de la leishmaniasis cutánea en pacientes pediátricos inmunosuprimidos, especialmente en un entorno con barreras sociales, económicas, y geográficas para el acceso a los servicios de salud. Dichas barreras pueden haber contribuido a la demora en el diagnóstico, así como a las interrupciones del tratamiento, y falta de seguimiento adecuado.

En conclusión, se debe considerar a la leishmaniasis en el diagnóstico diferencial cuando se atienda ulceras crónicas dermatológicas atípicas; incluso en aquellas que presentan un patrón diseminado en diferentes áreas anatómicas, especialmente en sujetos inmunosuprimidos que presenten sospecha epidemiológica. Asimismo, cabe resaltar la importancia de la identificación de la especie de *Leishmania* involucrada especialmente en aquellas formas agresivas o atípicas de la enfermedad, información que puede permitir el pronóstico de la evolución de la enfermedad, así como, un manejo clínico personalizado. Finalmente, es importante resaltar la efectividad terapéutica de la anfotericina B liposomal en pacientes inmunocomprometidos que requieren un tratamiento más agresivo y prolongado que el usual.
